# A review of the manufacturing process and infection rate of 3D-printed models and guides sterilized by hydrogen peroxide plasma and utilized intra-operatively

**DOI:** 10.1186/s41205-020-00061-w

**Published:** 2020-03-30

**Authors:** Graham Ka-Hon Shea, Kenneth Lap-Kei Wu, Iris Wai-Sum Li, Man-Fai Leung, Ada Lai-Ping Ko, Lane Tse, Sherby Suet-Ying Pang, Kenny Yat-Hong Kwan, Tak-Man Wong, Frankie Ka-Li Leung, Christian Xinshuo Fang

**Affiliations:** 1 Department of Orthopaedics and Traumatology, The University of Hong Kong, Hong Kong, Hong Kong; 2 School of Biomedical Sciences, The University of Hong Kong, Hong Kong, Hong Kong; 3 School of Public Health, The University of Hong Kong, Hong Kong, Hong Kong; 4 Department of Surgery, The University of Hong Kong, Hong Kong, Hong Kong; 5 Tam Shiu Foundation Anatomical Modelling Laboratory, Hong Kong, Hong Kong

**Keywords:** 3D printing, Infection, Sterilization, ABS

## Abstract

3D printing in the context of medical application can allow for visualization of patient-specific anatomy to facilitate surgical planning and execution. Intra-operative usage of models and guides allows for real time feedback but ensuring sterility is essential to prevent infection. The additive manufacturing process restricts options for sterilisation owing to temperature sensitivity of thermoplastics utilised for fabrication. Here, we review one of the largest single cohorts of 3D models and guides constructed from Acrylonitrile butadiene styrene (ABS) and utilized intra-operatively, following terminal sterilization with hydrogen peroxide plasma. We describe our work flow from initial software rendering to printing, sterilization, and on-table application with the objective of demonstrating that our process is safe and can be implemented elsewhere. Overall, 7% (8/114 patients) of patients developed a surgical site infection, which was not elevated in comparison to related studies utilizing traditional surgical methods. Prolonged operation time with an associated increase in surgical complexity was identified to be a risk factor for infection. Low temperature plasma-based sterilization depends upon sufficient permeation and contact with surfaces which are a particular challenge when our 3D-printouts contain diffusion-restricted luminal spaces as well as hollows. Application of printouts as guides for power tools may further expose these regions to sterile bodily tissues and result in generation of debris. With each printout being a bespoke medical device, it is important that the multidisciplinary team involved in production and application understand potential pitfalls to ensuring sterility as to minimize infection risk.

## Background

Advances in 3D printing has allowed for this technology to become increasingly prevalent across medical disciplines. In the context of facilitating surgical management, 3D-printed models can be an essential component of both pre-operative planning as well as intra-operative execution. Its main advantage lies in the capacity to display each patient’s unique anatomy, thus allowing for more accurate surgical procedures, often with a corresponding decrease in operative time and reduction in morbidity. Fused deposition modelling (FDM) is the most common and least costly printing technique and utilizes thermoplastic filaments as printing materials by means of extrusion from a heated print nozzle onto a build platform. In the specialty of orthopaedics, 3D models can allow for visualization of bony anatomy and implant contouring, whilst guides can be created to direct osteotomies as well as screw entry sites [1].

As 3D models/guides intended for intra-operative application can come into direct contact with patient tissues, it is critical that sterility is ensured [2]. Polymers with melting temperature below 132^∘^C are not compatible with steam sterilization as this would result in deformation [2, 3]. Low temperature (65^∘^C) sterilization methods suitable for 3D printouts include ethylene oxide (EtO) gas and hydrogen peroxide plasma, with the latter technique preferred due to its absence of toxic by-products [4]. Hydrogen peroxide plasma is generated following excitation beyond the gaseous phase and achieves sterility by inducing free-radical formation [5].

A multidisciplinary team at our orthopaedic academic unit has been producing 3D models and guides in-house since 2015. This was initially confined to orthopaedic applications but we have since expanded to provide printouts for other surgical contexts. A total of 300 models have been rendered over the past four years, with 114 cases confirmed to have been utilized intra-operatively. For such cases, it is not reported in the literature whether terminal sterilization by means of hydrogen peroxide gas plasma is sufficient in preventing infection [6]. Here, we review details for cases requiring the application of 3D models and guides intra-operatively, identify cases subsequently developing surgical site infection, and identify risk factors for infection. Biocompatibility of finished models and guides was not considered for this study. In detailing the design, printing, and sterilization of 3D printouts as well as infection-related outcomes amongst this sizable cohort, we demonstrate that our production process is safe for continuation and may be adopted elsewhere.

## Methods

### Patient identification and inclusion / exclusion criteria

**Fig. 1:**
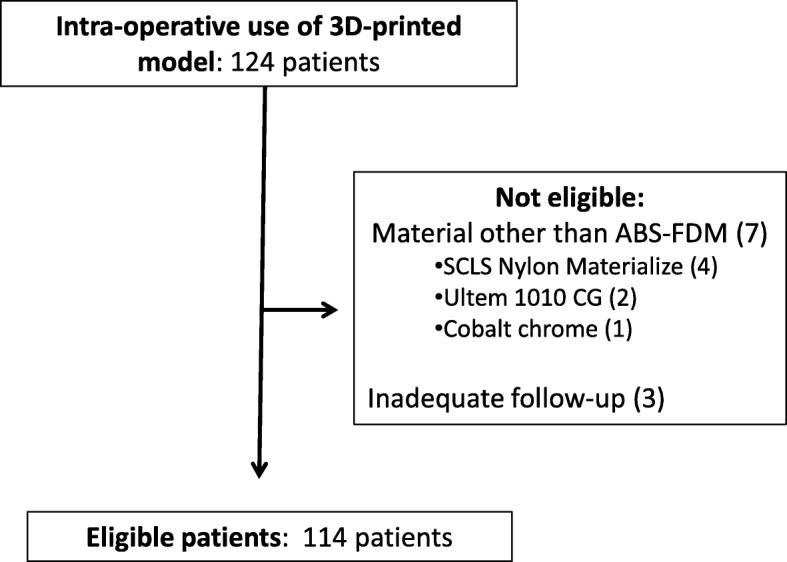
Patient eligibility and exclusion. More than 300 models were rendered by computer software from 2015 – 2019. The numbers of models proceeding to manufacturing, sterilization and intraoperative use amounted to 124. A further ten patients were excluded from analysis due to use of materials other than ABS (7 patients) as well as failure to reach 3-months of follow-up subsequent to surgery (3 patients), leaving a total of 114 patients eligible for analysis

We documented all 3D-printed models originating from our centre that were designed, printed, and utilized intra-operatively between the period of January 2015 and June 2019. Three sources of records from our 3D printing centre, TSSU (Theatre Sterile Supplies Unit) and electronic clinical notes were cross-referenced to ensure for intra-operative use and to clarify the details of application. Exclusion was on the basis of models not being utilized intraoperatively / insufficient documentation with regards to their use, <3 months follow-up from the index operation and of models not utilizing ABS as the printing material (Fig. 1).

Patient demographics (age, sex), comorbidities (diabetes mellitus, active malignancy), pre-operative serum albumin and operation time with regards to eligible cases were retrieved from electronic patient records. Cases of post-operative infection were identified via reporting from the responsible operating surgeon and from electronic patient records.

### 3D printing, processing and sterilization

**Fig. 2:**
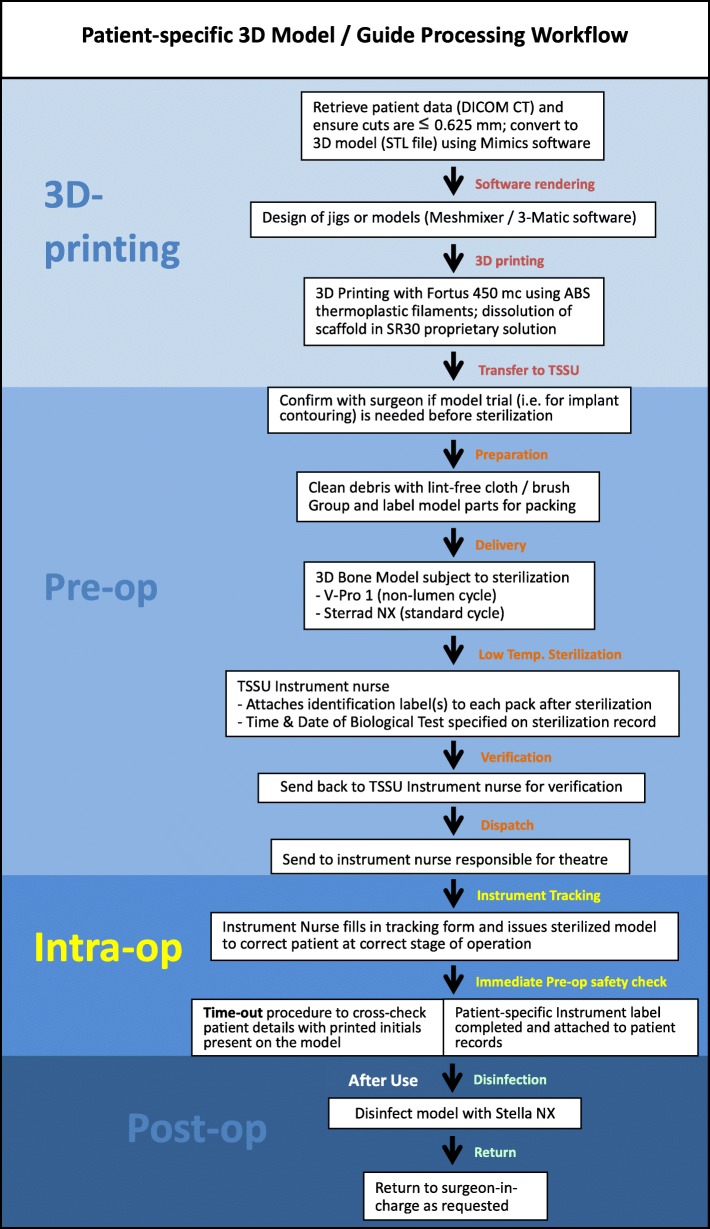
Patient-specific 3D model / guide workflow. Overview of the model / guide production process from design to printing, sterilization, and intra-operative usage. DICOM = Digital Imaging and Communications in Medicine; STL = stereolithography (file format); TSSU = Theatre Sterile Supplies Unit

**Fig. 3:**
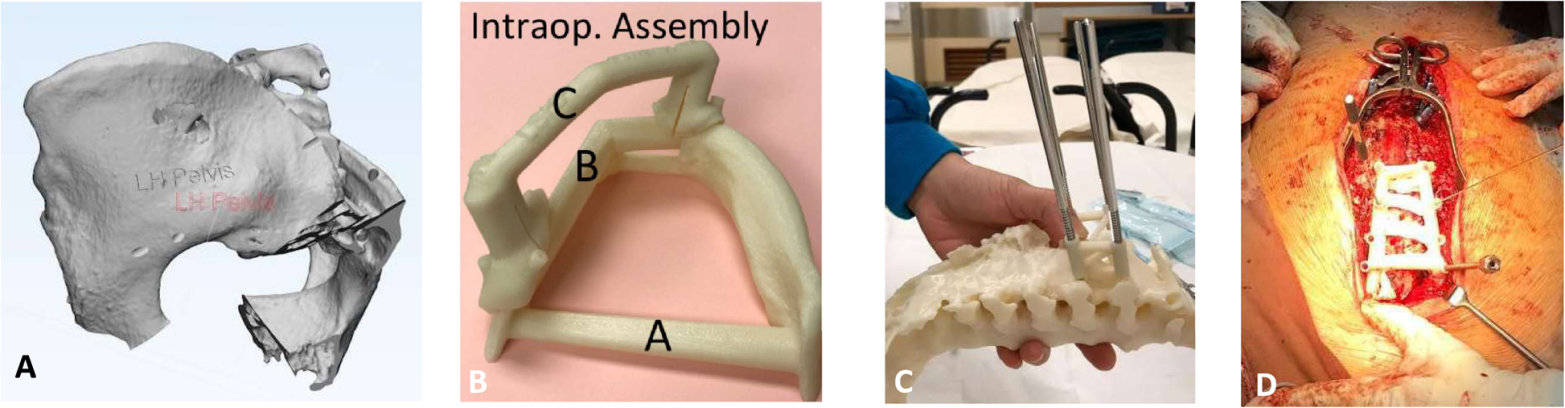
Aspects of model / guide manufacturing unique to intra-operative usage. **a** 3D-rendering of pelvis model with initials engraved upon the left ilium to allow for correct patient identification. **b** Photos taken by instrument nurse demonstrating proper grouping and assembly of a surgical guide for pedicle screw placement so corresponding components may be packaged and sterilized together. The assembled guide was contoured to fit upon bony surface landmarks of the posterior spinal vertebra, as demonstrated during testing upon a 3D-model of the same patient **c** and during the definitive surgery (**d**)

An overview of the manufacturing process of 3D-printed models is shown in Fig. 2. Digital imaging and communications in medicine (DICOM) based patient data of CT scans for the relevant bony region at 0.625mm or finer slice thickness were recovered, segmented and processed by identification of relevant anatomical landmarks. These were subsequently converted into a digital 3D model in the stereolithography (STL) format using Mimics software version 20 or 21 (Materialise, Leuven Belgium). 3D models and guides were designed with Meshmixer (Autodesk, USA) and 3-Matic software (Materialise, Belgium). We added the corresponding patient’s initials to non-essential regions of the printout to allow for on-table identification (Fig. 3a). The models and guides were manufactured with the FDM 3D printer Fortus 450mc (Stratasys, MI, USA) using the material ABS-M30i. Water soluble supporting material (SR30) utilized during the printing process was dissolved with WaterWorks^TM^ dissolution mixture at 70^∘^C for up to 24 hours.

Completed models / guides were cleansed with a lint-free cloth / brush to remove loose debris and rinsed with tap water. Printouts were air-dried before being packaged and subject to terminal sterilization. In the instance of models / guides with multiple components requiring assembly, the instrument nurse carefully photographed relevant groupings (Fig. 3b-d). Thereafter, grouped components were placed in labelled transfer boxes. Reprocessing records containing corresponding labels facilitated tracking of box contents. These steps enabled for relevant components to be packaged and sterilized together, and delivered to the surgeon intra-operatively in the correct combination and appropriate stage of surgery.

Printouts were sterilized with hydrogen peroxide plasma using either the V-Pro 1 Low Temperature Sterilization system (STERIS Corporation, Mentor, OH), or Sterrad $\circledR $ System (Advanced Sterilisation Products, Irvine, CA). Low operating temperatures in both systems prevented material deformation during sterilization. For the V-Pro 1 Low Temperature system, we selected the pre-programmed non-lumen cycle. For the Sterrad $\circledR $ NX system we selected the pre-programmed standard cycle. Sterilized models / jigs were sent back to the instrument nurse for verification, stored until use, and then passed to the surgeon during the corresponding operation session. A surgical time-out procedure ensured that the printout was used for the correct patient, anatomical region and procedure, in accordance to initials upon the surface (Fig. 3a). Post-operatively, printouts were similarly subject to low temperature disinfection then returned to the surgeon in charge.

### Statistics

Descriptive statistics were employed, with continuous variables expressed as mean ± standard error of the mean (SEM). With regards to tests of statistical significance, Fisher’s exact test was used for comparison of categorical data, Student’s T-test for comparison of continuous data between two groups, and ANOVA with post-hoc Tukey’s test for multiple groups of continuous data. A *p*-value <0.05 (one-tailed) was taken to demonstrate statistically significant difference between groups.

## Results

### Eligible cases and mode of application

**Fig. 4:**
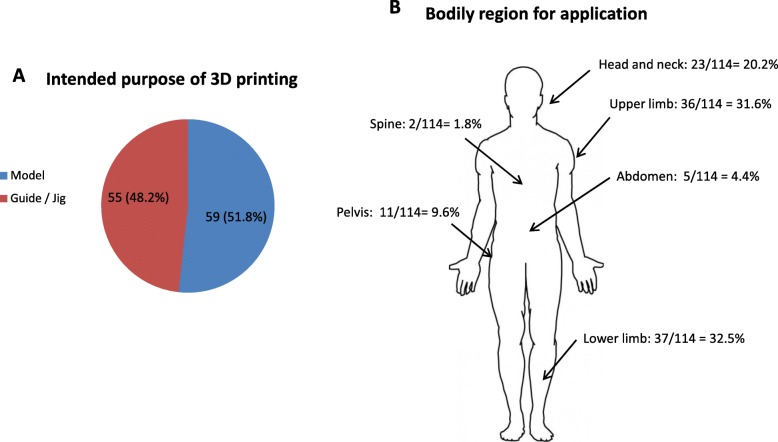
Application of 3D printouts. **a** Intended purpose of 3D printout showing 59/114 (51.8%) of printouts being utilized as anatomical models and 55/114 (48.2%) as guides / jigs intra-operatively. **b** The 124 cases utilized intraoperatively spanned different regions of the body as well as surgical specialties. The numbers relevant to each region and their percentages in relation to the whole patient cohort is shown

A total of 124 patients had 3D printouts that were utilized intra-operatively as part of their surgical management between the period of January 2015 and June 2019 (Fig. 1). Seven cases were excluded as printouts were not constructed from ABS, of which four cases utilized nylon, two case utilized polyetherimide (Ultem1010 CG), and one case utilized cobalt chrome. Three cases were excluded because of inadequate follow-up. A total of 114 models remained for subsequent analysis. Fifty nine out of 114 (51.8%) were anatomical models utilized on-table for planning and / or implant contouring (Fig. 4A). The remaining 55/114 (48.2%) were utilized as guides or jigs specific to patient anatomy to facilitate corrective osteotomies, screw insertion or pin placement. With regards to infection rate (Table 1A), 6/55 guides or jigs (10.9%) and 2/59 models (3.3%) developed surgical site infections, which was not a statistically significant difference (*p*=0.1141). All six cases of guides / jigs with infection were utilized to facilitate osteotomies. Both models with infection were utilized for implant contouring, one during fixation of a pilon fracture and the other for an orbital floor blowout fracture.

**Table 1:** (A) Table showing numbers of patients with infection according to mode of application as models or guides / jigs, which did not demonstrate statistically significant difference (p = 0.1141). (B) Infection rates according to bodily region of application, with numbers applied as models or guides / jigs in italics

A				
Mode of application	Infected cases	Non-infected cases	Total cases	Cases with infection (%)
Models	2	57	59	3.4
Guide / jig	6	49	55	10.9
B				
Region	Infected cases *(Models / Guides or jigs)*	Non-infected cases *(Models / Guides or jigs)*	Total cases	Cases with infection (%)
Head & Neck	4 *(1/3)*	19 *(9/10)*	23	17.4*
Upper limb	0	36 *(23/13)*	36	0
Lower limb	4 *(1/3)*	33 *(23/10)*	37	10.8
Pelvis	0	11 *(7/4)*	11	0
Abdomen	0	5 *(0/5)*	5	0
Spine	0	2 *(0/2)*	2	0

* p = 0.0195 with regards to a significantly elevated infection rate of head and neck vs. upper limb cases

### Bodily region of involvement and operative details

With reference to the bodily region that models / guides were utilized upon (Fig. 4B), 37/114 cases (32.5%) involved the lower limb, 36/114 models (31.6%) involved the upper limb, 23 cases (20.2%) were in the head and neck region, 11 cases (9.6%) in the pelvic region, five cases (4.4%) as percutaneous soft tissue guides over the abdomen, and two cases (1.8%) involved the spine. Surgical site infections (SSI) developed over the head and neck region (4/23; 17.4%) and lower limb (4/37; 10.8%) but were absent in the other bodily regions. In performing statistical comparison between infection rates of operations in these respective bodily regions (Table 1B), there was a significant increase in head and neck cases with infection as compared to upper limb cases (4/23, vs. 0/36; *p*=0.0195). The most common surgical procedures requiring three-dimensional printouts together with their mode of application, infection rate and average operation times are listed in Table 2. The operative time for patients receiving mandible resection was significantly higher than those receiving reverse shoulder arthroplasty, pilon fracture ORIF, and tibial plateau ORIF (*p*<0.01) although there was no statistical significant difference between infection rates (*p*=0.2792).

**Table 2:** The five most common applications amongst our patient cohort are listed, together with their infection rate, need for re-operation, and average length of operation in minutes (mean ± SEM)

Operation performed	Mode of application	Number of cases	Cases with infection (%)	Need for re-operation	Average operation time (minutes ± SEM)
Reverse shoulder arthroplasty	Guide	14	0	N/A	169 ± 17
Mandible resection	Guide	10	2 (20%)	1 of 2	649 ± 52*
Pilon fracture ORIF	Model	9	1 (11.1%)	0 of 1	193 ± 17
Acetabular / pelvis fracture ORIF	Model	7	0	N/A	400 ±159
Tibial plateau ORIF	Model	6	0	N/A	122 ± 23

* Patients receiving mandible resection had significantly longer operation time than those receiving reverse shoulder arthroplasty, pilon fracture ORIF, and tibial plateau ORIF (p < 0.01). ORIF = open reduction and internal fixation

With regards to the details of the 37 cases involving the lower limb, 24/37 (64.9%) were models used for intra-operative planning and/or plate contouring. These models were designed to facilitate surgical fixation of pilon fractures (nine patients), tibial plateau fractures (six patients), calcaneus fractures (four patients), ankle fractures (two patients), femoral shaft malunion (one patient), distal femur malunion (one patient) and metatarsal giant cell tumour (one patient). There were also 13 osteotomy guides (35.1%) printed for use over the lower limb. Three guides were based around the knee, to address deformity subsequent to fracture malunion (two patients) and as a complication of tuberculosis (one patient). Guides facilitated osteotomies over the distal femur in two patients suffering from osteosarcoma. Four patients had tibial shaft osteotomy guides, correcting for fracture malunion (two patients), rickets (one patient), and pseudoarthrosis secondary to neurofibromatosis (one patient). There was one patient each receiving corrective osteotomies for malunions of the fibula, tibial plateau, midfoot and calcaneus.

For the 36 cases involving the upper limb, 13 (36.1%) were models generated to facilitate intra-operative planning and/or plate contouring following fractures; of these seven were over the distal radius, two involved the mid-forearm, two involved the distal humerus, one involved the superior shoulder suspensory complex and one was over the proximal humerus. Another 23 (63.9%) were guides, which in 14 patients facilitated glenoid guidepin and screw placement during reverse shoulder arthroplasty. The remaining nine printouts were utilized as osteotomy guides. Of these, five were to address malunion of the distal radius, three for malunion of the distal humerus, and one to guide resection of the clavicle in a patient with malignant fibrous histiocytoma.

Over the head and neck region the most common application of 3D-printing was to generate mandible osteotomy guides in the management of malignancies, accounting for 10/23 cases (43.5%). Seven contralateral mirror-imaged auricular models were generated and brought on-table to facilitate microtia surgical reconstruction, as well as two orbital wall models for implant contouring during fixation of blow-out fractures. Two models allowed for plate contouring following mandibular fractures. One guide was generated to facilitate burring of excess bone in a case of fibrous dysplasia affecting the maxilla. Another guide facilitated cranioplasty in the management of a skull osteoma.

A total of 11 printouts were located over the pelvic region. Specifically, seven models (63.6%) were utilized for surgical planning, reduction and/or plate contouring for complex fractures affecting the pelvis and acetabulum. There were three cutting guides based around the pelvis, allowing for resection of acetabular metastasis, to facilitate conversion of a fused hip to arthroplasty, and for acetabular reshaping subsequent to malunion. There was also one screw insertion guide for sacroiliac fixation. Finally, five soft tissue guides were designed according to the patient’s abdominal contour for cryoablation of renal cell carcinoma, and two cutting guides to the spine were fashioned for vertebral column resection in the context of scoliosis and Schuermann’s kyphosis.

### Risk factors for infection

A total of 8/114 patients (7.0%) had SSIs subsequent to intra-operative use of 3D printouts. A comparison of patient demographics and risk factors between cases with and without infection is shown in Table 3; 20 patients were excluded from analysis due to insufficient information upon patient records. The average age amongst patients with infection was lower than that of non-infected cases (45.3 vs 53.7 years old; *p*=0.0348). Amongst the eight cases of infection there were six males and two females and gender was not a significant risk factor (*p*=0.197). The average operation time amongst cases with infection was 399±105 minutes as compared to 241±24 minutes, which was significantly longer (*p*=0.0373). As a reflection of case complexity, 4/8 cases with infection extended beyond 5 hours of operation time. With regards to co-existing comorbidities and nutritional status, neither diabetes mellitus (*p*=0.200), active malignancy (*p*=0.094) nor pre-operative albumin levels (*p*=0.151) demonstrated statistically significant differences between infected and non-infected cases.

**Table 3:** Demographics and risk factors comparing infected and non-infected cases

Demographics / Risk factors	Infected cases	Non-infected cases	*P*-value
Age	45.3 ± 3.7	53.7 ± 2.1	**0.0348**
Sex	Male – 6 Female - 2	Male – 45 Female – 41	0.197
Operation time (minutes)	399 ± 105	241 ± 25	**0.0373**
Diabetes mellitus	Yes – 3 No – 5	Yes – 16 No – 70	0.200
Active malignancy	Yes – 3 No – 5	Yes – 11 No – 75	0.094
Serum albumin (g/L)	37 ± 3.0	41.3 ± 0.5	0.151

Statistical comparison was carried out between relevant patient demographic details and risk factors for infection. Twenty cases were excluded from analysis due to incomplete medical records. With regards to analysed factors, younger age as well as longer operation times were identified as statistically significant risk factors for infection. Sex, presence of diabetes mellitus, active malignancy and serum albumin (obtained from pre-operative liver function tests) were not correlated with infection risk

Details for individual cases with infection are shown in Table 4. With regards to timing of presentation, 4/8 cases presented within 6-weeks of surgery. Cases presenting within the first week post-op included a case of periorbital cellulitis subsequent to orbital floor fixation that resolved with antibiotics, and a case of pilon fracture fixation that presented with persistent post-op fever due to an infected hematoma that required debridement. Cases of late infection following more than 6-months post-operation presented as a superficial infection (cellulitis) for two patients and as a deep infection (osteomyelitis) for one patient. The case of late infection due to osteomyelitis was the most serious amongst those reported within our patient cohort. This patient had a history of diabetes mellitus and neurofibromatosis and received a corrective wedge osteotomy of the tibia and fibula followed by both internal and external fixation to confer additional stability. The patient presented at 7-months post-operation and required multiple episodes of soft tissue and bone debridement for an infection involving the pin tracts tracking down to the tibia. Revision fixation and bone grafting of the resultant bony defect was required for reconstruction subsequent to debridement. Overall, surgical debridement and/or implant revision was required in 5/8 (62.5%) patients with infection.

**Table 4:** Details of cases with infection

Age / Sex	Diagnosis	Comorbid conditions	OT performed	Bodily region	OT time (min)	Application of 3D printing	Details	Need for re-operation	Micro-organism
37/M	Right orbit blowout fracture	Good past health	ORIF	H&N	67	Medphor implant contouring	Periorbital cellulitis noted on post-op day 2	No; resolved with antibiotics	Unknown
45/M	Right distal tibia chondroblastic osteosarcoma	DM, HT, polycystic kidney	Partial ostectomy of tibia / fibula, intramedullary nailing to tibia with bone transport, ORIF to fibula	H&N	603	Cutting guide for tibia and fibula osteotomy	Non-healing wound over right shin	Yes; debridement and closure	*MRSA, diphtheroid*
43/F	Left tibia fracture malunion	Borderline personality	Transverse tibial osteotomy and derotation	LL	127	Cutting guide for tibia osteotomy	Broken screws of medial plate noted 6-weeks after surgery with revision done; wound infection after revision	Yes; debridement	*MSSA*
69/F	Tuberculosis of right knee	DM, HT, scoliosis	Femoral osteotomy for correction of extraarticular deformity, total knee replacement, gastrocnemius flap, skin grafting	LL	876	Cutting guide for femoral osteotomy	Non-healing superficial knee wound with debridement 5-weeks post-op	Yes; debridement and negative pressure therapy	*MRCNS, candida parapsilosis*
41/M	Left tibial pseudoarthrosis	DM, NF type-I, hyperlipide mia	Wedge osteotomy of tibia and fibula with ORIF, application of external fixator	LL	239	Cutting guide for tibia and fibula osteotomy	Deep infection noted 7–months post-op with infection of pin tracts and osteomyelitis.	Yes; multiple episodes of surgical debridement, implant removal and application of temporary external fixator	*Streptococcus agalactaie*
80/M	Carcinoma of right tongue and alveolus	Good past health	Mandibulectomy with fibula graft reconstruction, pectoralis major flap	H&N	354	Cutting guide for femoral osteotomy, plate contouring	Fungal infection over pectoralis major flap noted 6-months post-op	No; resolved with topical and oral antifungals	*Candida albicans*
38/M	Carcinoma of right lower gum	Mallory-Weiss syndrome	Mandibulectomy with fibula flap reconstruction, modified radical neck dissection, selective neck dissection	H&N	715	Fibula graft resection guide	Presentation of cellulitis 6-months post-op	No; resolved with antibiotics	Unknown
41/M	Left pilon fracture	Good past health	ORIF	LL	210	Plate contouring	Persistent fever and postop with unhealthy hematoma and tissue noted upon debridement 1-week post-op	Yes; debridement and hematoma removal	*Burkholderia cepacia*

Details with regards to the eight patients amongst our cohort that developed infection. DM = diabetes mellitus, H&N = head and neck, HT = hypertension, LL ? lower limb, ORIF = open reduction internal fixation, MSSA = methicillin-sensitive staphylococcus aureus, MRSA = methicillin-resistant staphylococcus aureus, NF = neurofibromatosis

## Discussion

We have designed and manufactured a large number of 3D printouts with their intra-operative application ranging from models for plate contouring to corrective osteotomy guides and jigs for screw placement. With printouts in direct contact with patient tissues, it is important that we are able to assess for the risk of infection [6]. The overall infection rate of 7% was comparable to figures in the literature using traditional surgical techniques. Our process for the production of models and guides for intra-operative application is safe, yet users need to be aware of potential caveats.

Additive manufacturing utilizing thermoplastic filaments may in itself ensure for sterility of the immediate printout as biomaterials are subject to temperatures in excess of 200^∘^C [7]. Nevertheless a substantial amount of post-print processing is required, during which printouts are manipulated in non-sterile environments and therefore subject to contamination. Bacterial colonization resulting in biofilm formation upon ABS has been demonstrated [8], which enables for evasion from anti-microbials as well as host defences [9]. Roughness and topography are surface properties that influence biofilm formation, and of relevance to our printouts which reproduce irregular bony surfaces [10]. We have on occasion stored our printouts post-sterilization for weeks, or conversely proceeded to intra-operative utilization without a sufficient quarantine period post-sterilization, both of which may predispose to infection. Nevertheless formal regulation of design, manufacturing and sterilization processes will be difficult as each printout is a unique patient-specific device [11]. Use of biological indicators can be a means of supporting adequate sterilization for each customized printout.

**Fig. 5:**
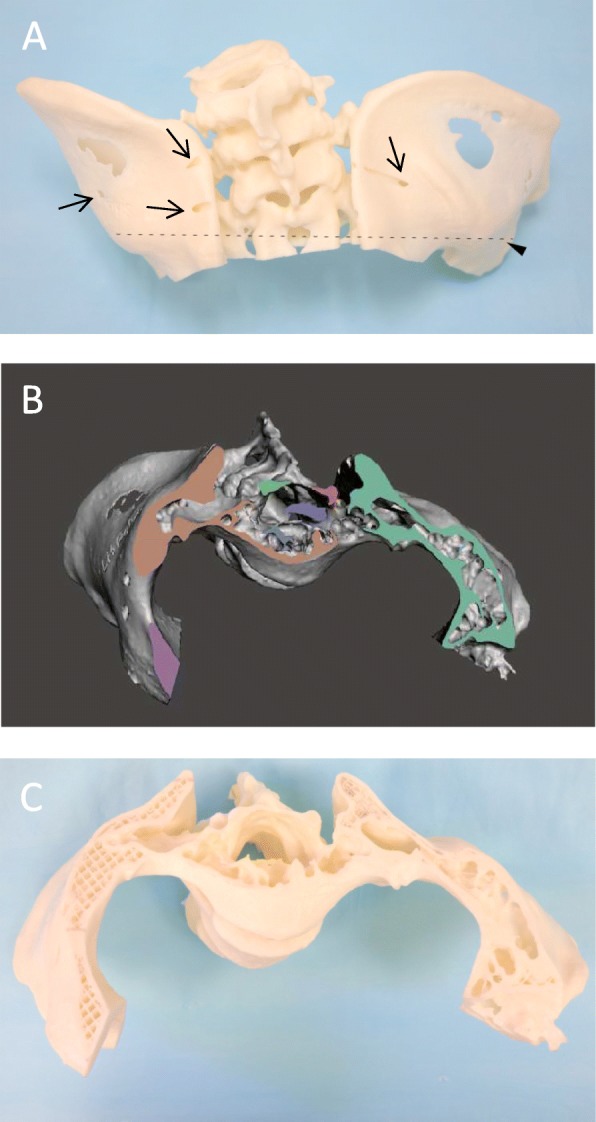
Cross-section of pelvis 3D model demonstrating irregular luminal spaces. **a** Arrows indicate surface openings upon the posterior ilium on a 3D model of the pelvis. The dotted line and arrowhead demonstrates the level of transverse sectioning subsequently performed. Cross-sectional appearance following software rendering **b** and physical sectioning **c** of the same pelvis model demonstrating irregular trabecular spaces contained within

We predominantly used the V-Pro 1 low-temperature sterilization system for our heat-sensitive 3D printouts. This system utilizes hydrogen peroxide plasma to achieve sterility and is applicable across a range of heat- and moisture-sensitive materials. Sterilization is dependent upon plasma permeation and direct contact, with confined spaces involving long narrow lumens, for example during the sterilization of endoscopes, being potentially problematic. Under controlled testing however, plasma sterilization eradicated bacteria spores seeded to a depth of 500 mm within 0.7 mm diameter tubing [12]. It is uncertain how hydrogen peroxide plasma will penetrate into narrow lumen (Fig. 5a) leading to irregular trabecular spaces (Fig. 5b and c) that correspond to cancellous bony structure. Many of these spaces are enclosed and do not directly communicative with the external environment to allow for gas permeation and risks contamination subsequent to intra-operative exposure. Biological indicators may be embedded within the complex internal structure as a form of stress testing. Alternately in being non-essential for function, internal structural motifs could be simplified in design prior to printing. Nevertheless, plasma based methods would be advantageous for the permeation of internal structures in comparison to surface sterilization of heat-sensitive materials by ultraviolet light [13]. Seperately, removal of supporting material by heating under alkali conditions may be incomplete [14] thus shielding ABS from sterilization.

**Fig. 6:**
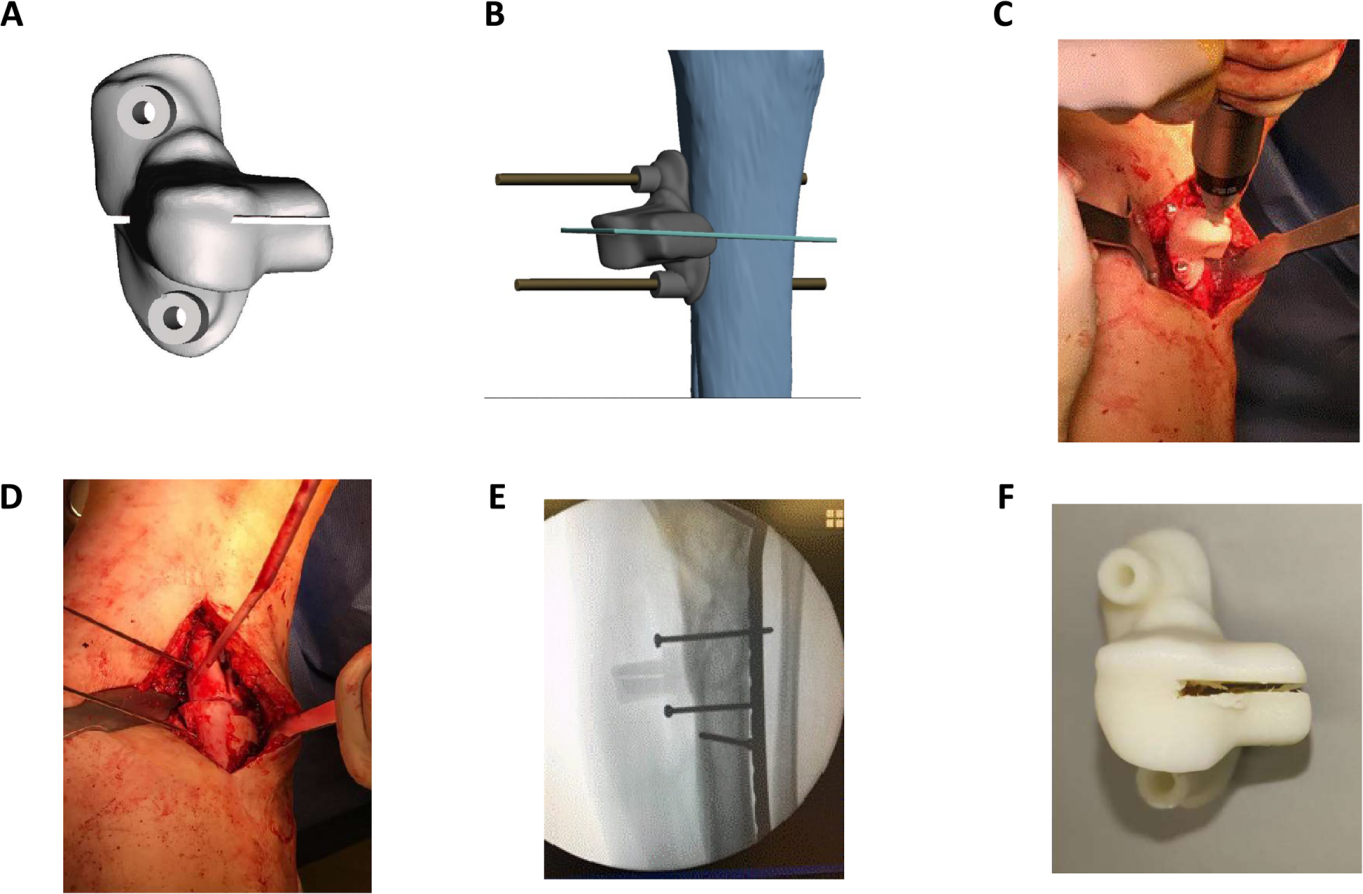
Intraoperative use of osteotomy guide. **a** Software rendered image of guide intended for corrective osteotomy and shown upon the tibial shaft (**b**) with the osteotomy site marked in teal. **c** Intra-operative photo with the guide secured and oscillating saw engaged in preparation for osteotomy and (**d**) upon completion. **e** Intra-operative x-ray demonstrating reduction and fixation of the tibial shaft following corrective osteotomy. **f** Similar guide retrieved post-op demonstrating damage to ABS over the osteotomy slit with the potential to release debris

Tissue biocompatibility is an additional factor that merits discussion. Due to contact between exposed tissues and printouts as well as debris generated by intra-operative damage (Fig. 6) it is important to be able to ensure for the safety of such medical devices by biocompatibility testing. Developed by the International Organization of Standardization, ISO 10993 describes a series of standards for biocompatibility testing including cytotoxicity assays, assays for irritation and skin sensitisation, as well as guidelines for sample extraction. ABS-M30i complies with ISO 10993 standards yet this is with regards to its raw form prior to printing and post-print processing. Inspection of damage to guides / jigs, removal of visible fragments over the surgical site, and copious irrigation of exposed tissues would provide a simple means to reducing cytotoxicity and debris retention. We did see a higher proportion of infections in the application of printouts as guides / jigs as compared to models. Nevertheless this increase in infection rate could also be the result of lengthier and more complex operations, as we have demonstrated operative time to be a risk factor. The younger age of patients with infection as compared to those without was an unexpected finding, but likely due to the management decision for complex curative surgery and reconstruction amongst those suffering from malignancy when age and co-morbidities were not contraindications for a major operation.

With regards to the benefits of 3D modelling on surgical execution, a meta-analysis compiling studies for pilon fracture management reported that operation time, blood loss, post-operative pain scores, achievement of anatomical fracture reduction, and post-operative function were all improved in comparison to conventional planning [6]. Rates of infection following 3D modelling amongst this cohort were not increased, and have been reported elsewhere to be as high as 16.1% owing to the degree of associated soft tissue trauma, with an average presentation time of 88-days post-op [15]. Amongst out patients with pilon fractures, 1/9 (11.1%) developed infection. Another study utilized 3D-printed ABS models to facilitate fixation of complex tibial plateau fractures in 69 patients [16]. A total of 6/69 (8.7%) suffered from SSIs, whilst within our cohort none of the six cases did. Conventional reverse shoulder arthroplasty has a reported infection rate of approximately 5% [17], and amongst our cohort none of the 14 patients receiving this procedure in conjunction with 3D-printed guides for glenoid guidepin / screw placement had a surgical site infection within 3-months. Mandibular resection and reconstruction in the treatment of malignant tumours is a formidable challenge with complication rates approaching 70% [18] and in our cohort head and neck cases (4/23; 17.4%) represented the region with the highest infection rate. We did not see evidence in the cases with infection indicating the persistence of sterilization-resistant microorganisms such as bacterial spores [19] following hydrogen peroxide plasma treatment. It is worth noting that prior studies detailing infection-related outcomes of 3D printouts have not explicitly utilized them intra-operatively, and this is one of the first studies to have done as such. Our overall impression was that our process of sterilization and on-table usage is safe, and that surgical complexity and tissue manipulation as reflected by increased operating time were the main culprits for infection.

Limitations to this study included its retrospective design without a designated control group, and insufficient power for detailed subgroup analysis. Designating 3-months of follow-up as an inclusion criterion may have failed to account for infections presenting at a later phase. It would be of interest to report on an increased cohort size over a longer period of follow-up at our center as the process continues to mature, in order to account for these present deficiencies.

## Conclusion

We have described one of the largest patient cohorts to receive intra-operative application of 3D models / guides. Our present workflow for the manufacturing, sterilization and intra-operative application of 3D models is safe for continuation as well as implementation elsewhere. With each printout being a bespoke medical device, it is essential for users to be aware of the unique structural properties of models / guides as well as constraints in the production process with regards to infection and biocompatibility. We forsee that the scope of application during surgery can only expand once the production process is established to be reproducible, consistent and safe.

## Abbreviations

ABS: Acrylonitrile butadiene styrene
DM: Diabetes mellitus
FDM: Fused deposition modelling
EtO: Ethylene oxide
HT: Hypertension
MSSA: Methicillin-sensitive staphylococcus aureus
MRSA: Methicillin-resistant staphylococcus aureus
MRCNS: Methicillin-resistant coagulase-negative staphylococci
NF: Neurofibromatosis
ORIF: Open reduction internal fixation
SSI: Surgical site infection
TSSU: Theatre Sterile Supplies Unit
